# Bacterial endotoxin adhesion to different types of orthodontic adhesives

**DOI:** 10.1590/1678-7757-2016-0434

**Published:** 2017

**Authors:** Priscilla Coutinho ROMUALDO, Thaís Rodrigues GUERRA, Fábio Lourenço ROMANO, Raquel Assed Bezerra da SILVA, Izaíra Tincani BRANDÃO, Célio Lopes SILVA, Lea Assed Bezerra da SILVA, Paulo NELSON-FILHO

**Affiliations:** 1 Universidade de São Paulo, Faculdade de Odontologia de Ribeirão Preto, Departamento de Clínica Infantil, Ribeirão Preto, SP, Brasil.; 2 Universidade de São Paulo, Faculdade de Medicina de Ribeirão Preto, Departamento de Bioquímica e Imunologia, Ribeirão Preto, SP, Brasil.

**Keywords:** Corrective orthodontics, Composite resins, Endotoxins

## Abstract

Bacterial endotoxin (LPS) adhesion to orthodontic brackets is a known contributing factor to inflammation of the adjacent gingival tissues.

**Objective:**

The aim of this study was to assess whether LPS adheres to orthodontic adhesive systems, comparing two commercial brands.

**Material and Methods:**

Forty specimens were fabricated from Transbond XT and Light Bond composite and bonding agent components (n=10/component), then contaminated by immersion in a bacterial endotoxin solution. Contaminated and non-contaminated acrylic resin samples were used as positive and negative control groups, respectively. LPS quantification was performed by the Limulus Amebocyte Lysate QCL-1000™ test. Data obtained were scored and subjected to the Chi-square test using a significance level of 5%.

**Results:**

There was endotoxin adhesion to all materials (p<0.05). No statistically significant difference was found between composites/bonding agents and acrylic resin (p>0.05). There was no significant difference (p>0.05) among commercial brands. Affinity of endotoxin was significantly greater for the bonding agents (p=0.0025).

**Conclusions:**

LPS adhered to both orthodontic adhesive systems. Regardless of the brand, the endotoxin had higher affinity for the bonding agents than for the composites. There is no previous study assessing the affinity of LPS for orthodontic adhesive systems. This study revealed that LPS adheres to orthodontic adhesive systems. Therefore, additional care is recommended to orthodontic applications of these materials.

## Introduction

Orthodontic appliances are composed of different materials and accessories with irregular surfaces like brackets, ligatures, bands and wires that create additional sites that harbor dental plaque and oral microorganisms^21^, changing chemical properties of the oral medium^10^. Fixed orthodontic therapy inevitably predisposes patients to an increased risk of dental problems, as fixed appliances make an effective oral hygiene challenging and limit the mechanical cleansing of saliva flow, tongue and oral muscles^24^.

The use of orthodontic appliances can also increase the levels of periodontal pathogens in the supragingival and subgingival, associated with gingival inflammation that can occur during orthodontic treatment^14^.

Periodontopathogenic microbiota is predominantly composed of anaerobic microorganisms^9^, especially Gram-negative bacteria^18^, which contain endotoxin in their cell wall^23^. Bacterial endotoxin, also referred to as LPS due to its lipopolysaccharide nature, is released during bacterial multiplication or death, causing a series of important biological effects^23^ that lead to inflammatory reaction and bone resorption in the periapical region^25^.

Endotoxin has a high affinity for different materials, e.g., metals^13^, silica, zirconium^7^, acrylic resins^4^, ceramics^13^ and even titanium and titanium alloys^1^.

*In vitro* and *in vivo* studies^11,19^ have shown that bacterial endotoxin adheres to metal brackets and such affinity affects endotoxin concentration in the gingival sulcus, contributing to inflammation of tissues adjacent to the brackets. By analogy, a similar process could occur on the surface of adhesive systems used for fixation of orthodontic brackets to the dental enamel. To the best of our knowledge there is not a previous study assessing bacterial endotoxin affinity for orthodontic adhesive systems. Therefore, the aim of this study was to assess whether LPS adheres to the components of orthodontic adhesive systems (bonding agent and composite resin), comparing two commercial brands.

## Material and methods

### Fabrication of specimens

In order to obtain the test specimens, it was used a circular Teflon matrix, manufactured at the Precision Workshop of the University of São Paulo, Ribeirão Preto, SP, Brazil. The matrix consisted of two nested parts: an outer portion and an inner portion in the form of a 3-mm-diameter plunger. Accompanying the matrix there was a 2-mm-thick spacer, which was engaged in the plunger between the two portions so that the outer potion was 2 mm higher than the inner portion, providing adequate thickness to the specimen.

Therefore, forty disc-shaped specimens (3 mm diameter and 2 mm thick) were fabricated from each component (composite or bonding agent) of two largely used orthodontic adhesive systems (Transbond XT; 3M Unitek, Monrovia, CA, USA and Light Bond; Reliance Orthodontic Products, Inc., Itasca, IL, USA). Groups were created with 10 specimens of each component (test groups). As bacterial endotoxin is known to have a high affinity for acrylic resin^4^, 10 additional specimens of a self-curing acrylic resin (JET Classic; Art. Odontológicos Ltda, Campo Limpo Paulista, SP, Brazil) served as positive (n=5) and negative (n=5) controls; the positive control was contaminated with the endotoxin solution and negative control was not contaminated.

Each component was inserted into the matrix in increments followed by pressure with a glass plate until excess flow. All components were activated with a halogen light device for 40 seconds, with light intensity of 400 mW/cm^2^. Then, specimens were removed from the matrix and their size checked with a precision caliper.

All specimens were sterilized with ethylene oxide (Oximed, São José do Rio Preto, SP, Brazil) and then contaminated by immersion in a bacterial endotoxin solution, except for the negative control group.

### Endotoxin (LPS) solution preparation

In a laminar flow chamber, 350 mg of lyophilized endotoxin from *Escherichia coli* (*Lipopolysaccharide B E.coli* 055:B5 – Sigma Aldrich Corporation, St. Louis, MO, USA) was suspended into 4.7 mL of pyrogen-free water, resulting in a 25 ng/mL concentration endotoxin solution. For contamination, the specimens were immersed in the solution in glass tubes placed under agitation (126 rpm) in an incubator at 37°C for ٢٤ h. The negative control specimens were not immersed in the solution (negative control).

### Quantification of bacterial endotoxin (LPS) by the Limulus Amebocyte Lysate QCL-1000™ test

After contamination with LPS, the specimens were individually placed in new nonpyrogenic glass tubes with lids (BioWhittaker; Cambrex Corporation, East Rutherford, NJ, USA) containing 1 mL of pyrogen-free water (recovery solution) and taken to an ultrasonic cleaner (Ultracleaner USC 1600ª; Unique Indústria e Comércio de Produtos Eletrônicos Ltda., Indaiatuba, SP, Brazil) for 15 min to release endotoxin from the material.

Endotoxin quantification in the bonding agent, composite and acrylic specimens was performed using the QCL-1000™ test (Limulus Amebocyte Lysate QCL-1000™; Lonza, Walkersville, MD, USA) following the manufacturer’s instructions. LAL is a quantitative test for detection of endotoxin with a sensitivity range of 0.1 - 1.0 EU/ml (endotoxin units *per* milliliter). A standard curve of known endotoxin levels was used to determine the amount of endotoxin in the samples. Fifty µL of solutions of each known standard concentration (1.0 EU/mL, 0.5 EU/mL, 0.25 EU/mL and 0.1 EU/mL) and 50 µL of the negative control (pyrogen-free water) was dripped in duplicate in the wells of a non-pyrogenic 96-well polystyrene plate (Corning Incorporated, Corning, NY, USA). Fifty µl of the samples diluted in pyrogen-free water at a ratio of 1:1 were added to the remaining wells and after that 50 µL of LAL solution were added to all wells containing samples or standards, the microplate was then incubated at 37°C for 10 min. After that, 100 µL of chromogenic substrate that was preheated to 37°C was added to the wells, stirred and incubated at 37°C for 6 min in the dark, following the same dripping protocol and maintaining a constant dripping rate. Subsequently, 100 µL of the blocking reagent (25% v/v glacial acetic acid in water) was added to stop the reaction.

The absorbance of each sample was determined using an ELISA (enzyme-linked immunosorbent assay) reader (Ultramark; Bio-Rad Laboratories, Hercules, CA, USA) at 405 nm. Absorbance was considered directly proportional to endotoxin levels in the wells and it correlated directly to the endotoxin concentration in the range from 0.1 to 1.0 EU/mL. The amount of endotoxin in each sample was expressed in EU/mL and calculated from the solution absorbance values with known endotoxin levels (standard) multiplied by the dilution factor.

### Statistical analysis

For statistical analysis, values of endotoxin concentrations were classified into three scores: score 1 (concentration ≤0.5 EU/mL); score 2 (0.51 to 1.0 EU/mL); and score 3 (>1.0 EU/mL). Comparisons of scores between composites and bonding agents and between the two brands of both types of materials were performed with the Chi-square test, using the GraphPad 5.0a Software (Graphpad Software Inc., San Diego, CA, USA). The significance level was set at 5%.

## Results

All experimental groups differed significantly (p<0.05) from the negative control group (non-contaminated acrylic resin), demonstrating bacterial endotoxin adhesion to all tested materials. No statistically significant difference (p>0.05) was found between experimental groups and positive control group (acrylic resin contaminated with endotoxin).

**Figure 1:**
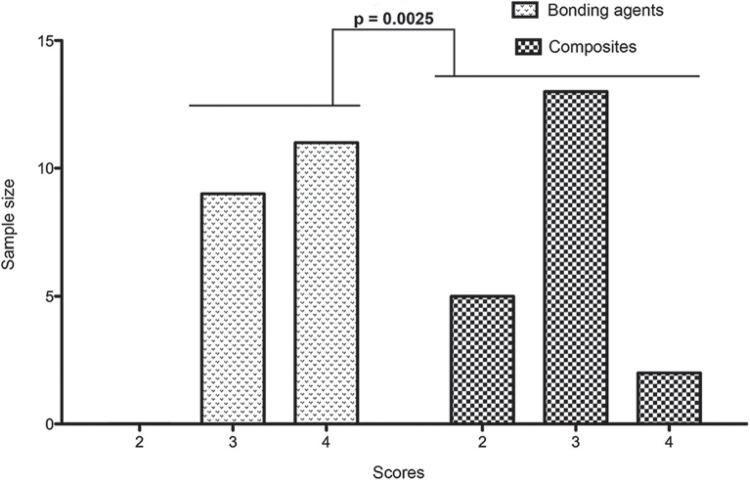
Score distribution of endotoxin concentrations for bonding agents and composites

Table 1 shows the distribution of relative and absolute endotoxin levels (by scores) in experimental groups. Since no statistically significant difference (p>0.05) was found between the two composites or the two bonding agents, the materials were compared regardless of their brand. Figure 1 is a graphical representation of scores distribution between composites and bonding agents, and it shows a significantly higher endotoxin adhesion to bonding agents than to composites (p=0.0025).

**Table 1:** Comparison of endotoxin concentration scores in experimental groups (composite and bonding agents)

Score	Transbond XT composite	Light Bond composite	p	Transbond XT bonding agent	Light Bond bonding agent	p
	**n (%)**	**n (%)**		**n (%)**	**n (%)**	
1	2 (20%)	3 (30%)	0.87	0 (0%)	0 (0%)	0.17
2	7 (70%)	6 (60%)		6 (60%)	3 (30%)	
3	1 (10%)	1 (10%)		4 (40%)	7 (70%)	

## Discussion

The present study quantified *in vitro* bacterial endotoxin adhered to the components (composite and bonding agent) of two orthodontic adhesives. The use of tests derived from the aqueous extract of *Limulus polyphemus* crab blood cells, known as Limulus Amebocyte Lysate (LAL) tests, is recommended to assess the presence or absence of endotoxin in solutions or instruments. In the presence of endotoxin, LAL components are activated by a protein cascade, which results in the cleavage of a substrate present in the test reagent, with the release of yellowish p-nitroaniline (pNA). The release of pNA is measured spectrophotometrically at 405-410 nm, after disruption of the reaction by a stop reagent. The LAL test has been widely used for endotoxin detection in different areas of Dentistry.

Bacterial endotoxin (LPS) is a major virulence factor of the surface of Gram-negative microorganisms, playing a key role in triggering periodontal inflammation^26^. It is a bacterial antigen present in the subgingival biofilm, acting directly on the innate immune system at the site of infection^27^. Acting as a powerful stimulus for a variety of host cells, LPS stimulates the expression of important pro-inflammatory cytokines, such as IL-1 and TNF-α^5^, which increase the expression and release of other pro-inflammatory cytokines and induce the release of cell adhesion molecules^8^. LPS also stimulates the production of reactive oxygen species and the phosphorylation of protein kinases in the cells^2^. In this way, LPS contributes to the recruitment of immune cells, a major component of the innate immune response^17^, causing a series of biological effects that trigger an inflammatory response with subsequent bone resorption^25^.

Endotoxin has high affinity for a variety of dental materials^1,7,13^, including acrylic resin commonly used as a temporary material^4^, and a high affinity for titanium (present in dental implants) with a significant decrease in titanium corrosion resistance^30^. Also in Dentistry, endotoxin is present in necrotic root canals^15^ where its presence has been associated with periapical inflammation and bone resorption^6^.

Our option of using endotoxin derived from *E. coli* in this study was based on its broad indication, based on its proven toxicity, to evaluate the biological activity of LPS at different research levels^12,25^. In addition, the molecular structure of *E. coli*, according to Mattison, et al.^16^ (1987), is representative of most endotoxins. Moreover, this endotoxin is easier to obtain and cheaper.

The results of this *in vitro* study showed that bacterial endotoxin has affinity for adhesives frequently used in orthodontics. Previous studies have demonstrated that LPS also adheres to metallic brackets, contributing to the inflammation of tissues adjacent to the brackets^11,19^. However, the lack of studies assessing endotoxin adhesion to other orthodontic adhesive systems does not allow to compare our findings.

Numerous orthodontic adhesive systems are available for bonding orthodontic brackets. The choice for Transbond XT is because it is often referred to a “gold standard” in a number of studies^28^. Light Bond adhesive system is also widely used in orthodontic practice and it was selected due to its fluoride releasing property. Fluoride-containing orthodontic adhesives have gained attention due to thebeneficial role of fluoride in inhibiting enamel demineralization around orthodontic brackets^20^.

Previous studies evaluating the same adhesive systems showed differences among them regarding shear bond strength^22^, degree of monomer conversion and cytotoxicity^3^. In the present study, however, the affinity of bacterial endotoxin for both materials was similar.

An important finding of the present study was the occurrence of greater bacterial endotoxin adhesion to bonding agents than to composites, which could be explained by differences in their composition. Although these materials have a similar composition, it is known that composites must contain higher amounts of inorganic filler particles, which are not always present in bonding agents^29^.

According to the manufacturers, the bonding agents evaluated in this study do not contain inorganic fillers, while both composites have over 80% of inorganic particles by volume.

Considering the higher affinity of endotoxin for orthodontic bonding agents, additional care is recommended to orthodontists in the sense of avoiding “overwetting” and limiting the application of these materials to the bracket base. Excess material on dental enamel should be carefully removed to avoid leaving areas of bonding agent/composite exposed to oral medium, which could favor the adhesion of LPS to the materials and stimulate the occurrence of inflammation in the gingival tissues adjacent to the brackets.

Further laboratory research and clinical studies are necessary to compare and substantiate these findings.

## Conclusion

The results of this study revealed that bacterial endotoxin (LPS) adhered to orthodontic adhesive systems. The bonding agents of both systems presented greater affinity for endotoxin than for composites.
